# Crosstalks between NOD1 and Histone H2A Contribute to Host Defense against *Streptococcus agalactiae* Infection in Zebrafish

**DOI:** 10.3390/antibiotics10070861

**Published:** 2021-07-15

**Authors:** Xiaoman Wu, Fan Xiong, Hong Fang, Jie Zhang, Mingxian Chang

**Affiliations:** 1 State Key Laboratory of Freshwater Ecology and Biotechnology, Key Laboratory of Aquaculture Disease Control, Institute of Hydrobiology, Chinese Academy of Sciences, Wuhan 430072, China; xiaomanwu1993@163.com (X.W.); xiongfan@ihb.ac.cn (F.X.); fanghong@ihb.ac.cn (H.F.); zhangjie@ihb.ac.cn (J.Z.); 2 Innovation Academy for Seed Design, Chinese Academy of Sciences, Wuhan 430072, China; 3 University of Chinese Academy of Sciences, Beijing 100049, China

**Keywords:** *Streptococcus agalactiae* infection, NOD1, histone, immune-related pathways, metabolic pathways

## Abstract

Correlation studies about NOD1 and histones have not been reported. In the present study, we report the functional correlation between NOD1 and the histone H2A variant in response to *Streptococcus agalactiae* infection. In zebrafish, NOD1 deficiency significantly promoted *S. agalactiae* proliferation and decreased larval survival. Transcriptome analysis revealed that the significantly enriched pathways in NOD1**^−/−^** adult zebrafish were mainly involved in immune and metabolism. Among 719 immunity-associated DEGs at 48 hpi, 74 DEGs regulated by NOD1 deficiency were histone variants. Weighted gene co-expression network analysis identified that H2A, H2B, and H3 had significant associations with NOD1 deficiency. Above all, *S. agalactiae* infection could induce the expression of intracellular histone H2A, as well as NOD1 colocalized with histone H2A, both in the cytoplasm and cell nucleus in the case of *S. agalactiae* infection. The overexpression of H2A variants such as zfH2A-6 protected against *S. agalactiae* infection and could improve cell survival in NOD1-deficient cells. Furthermore, NOD1 could interact with zfH2A-6 and cooperate with zfH2A-6 to inhibit the proliferation of *S. agalactiae*. NOD1 also showed a synergetic effect in inducing the expression of many antibacterial genes, especially antibacterial pattern recognition receptors *PGRP2*, *PGRP5,* and *PGRP6*. Collectively, these results firstly highlight the roles of NOD1 deficiency in the regulation of immune-related and metabolic pathways, and the correlation between zebrafish NOD1 and histone H2A variant in the defense against *S. agalactiae* infection.

## 1. Introduction

*Streptococcus agalactiae*, also known as Group B Streptococcus (GBS), is a Gram-positive bacteria, which infects a variety of hosts. Among them, the most commonly studied species are humans, cattle, and fish [1]. In humans, GBS is an important cause of neonatal meningitis, pneumonia, sepsis, and other severe invasive diseases in neonates and other infants, pregnant women, immunocompromised individuals, and older adults [2,3,4,5]. In teleost, streptococcal infections are responsible for global economic losses in farmed tilapia [6]. In addition to tilapia, *S. agalactiae* is also a major pathogen infecting other saltwater or freshwater fish, such as Javanese medaka *Oryzias javanicus* [7], giant Queensland grouper *Epinephelus lanceolatus* [8], cultured golden pomfret *Trachinotus blochii* [9], and olive flounder *Paralichthys olivaceus* [10].

In mammals and teleost fish, the innate immune response has been shown to play a vital role in controlling in vivo growth of *S. agalactiae*. Upon infection, the host innate immune system senses *S. agalactiae* by distinct classes of pattern recognition receptors (PRRs) such as Toll-like receptors (TLRs) and NOD-like receptors (NLRs) [11,12,13,14]. It has been found that *S. agalactiae* releases diacylated molecules to interact with TLR2/TLR6, which is critical for limiting bacterial dissemination and systemic inflammation in mice [15]. TLR13a and TLR13b from Nile tilapia can combine with the 23S rRNA of *S. agalactiae* [16]. *S. agalactiae* is also controlled by caspase-1-mediated innate immune response, which is activated by the assembly of the NLRP3 inflammasome, consisting of the sensor NLRP3, the adaptor-apoptosis-associated speck-like protein ASC, and the effector protein caspase-1. The mice lacking NLRP3, ASC, or caspase-1 were more susceptible to *S. agalactiae* infection than the wild-type mice [14]. Nucleotide-binding oligomerization domain-containing 1/2 (NOD1/NOD2) and NLRP3 are two groups of well-characterized NLRs. Different from NLRP3, NOD2 did not contribute to host defense against *S. agalactiae* in adult mice [17]. Whether NOD1 plays a major role in the pathogenesis of *S. agalactiae* remains to be resolved.

Five major classes of histones including H1, H2A, H2B, H3, and H4 are fundamental structural components of chromatin, which have been implicated in the regulation of chromatin remodeling, DNA repair, transcriptional activity, and individual development by histone posttranslational modifications [18,19]. Many pathogens such as *Listeria monocytogenes* have evolved to exploit epigenetic histone modifications and chromatin remodeling to modulate the host response during infection [20,21]. The antimicrobial activities of histones were also reported. Histones can act as antimicrobial peptides and directly kill bacteria, fungi, parasites, and viruses in a diverse range of organisms from shrimps to humans [22,23,24,25,26]. In addition, histones can trigger inflammatory responses acting through TLRs, including TLR2 and TLR4, which result in death in inflammatory injury [27]. Histones also activate the NLRP3 inflammasome during liver hepatic ischemia/reperfusion (I/R) through the TLR9-dependent generation of reactive oxygen species [28]. However, how histones and their crosstalks with NLRs contribute to host defense against *S. agalactiae* infection are still unclear.

Receptor-interacting protein 2 (RIP2) is a kinase that is involved in antibacterial signaling of NOD1/NOD2 sensing bacterial peptidoglycans. Our previous reports showed that NOD1 deficiency affected larvae survival in the early ontogenesis via CD44a-mediated PI3K-Akt signaling, which was independent of adaptor protein RIP2 [29,30]. To our surprise, RIP2 deficiency impaired the expression of all of the tested histones, including H2A, H2B, H3, and H4. The overexpression of histone H2A demonstrated an increased survival rate of zebrafish larvae infected with *Edwardsiella piscicida*, and an increased transcription of many antibacterial genes. The transcription regulations of histone H2A on these antibacterial genes were dependent on RIP2, and no significant changes, even decreased expressions, were observed for these antibacterial genes in RIP2*^−/−^* zebrafish [31]. In this work, we firstly describe the functional characterization of NOD1 and the histone H2A variant in response to Gram-positive bacteria *S. agalactiae* in vitro and in vivo. More importantly, our results reveal that the interaction and cumulative effect exist between NOD1 and the histone H2A variant for protecting against *S. agalactiae* infection.

## 2. Results and Discussion

### 2.1. NOD1 Contributes to Host Defense against S. agalactiae Infection in Adult Zebrafish

**Figure 1:**
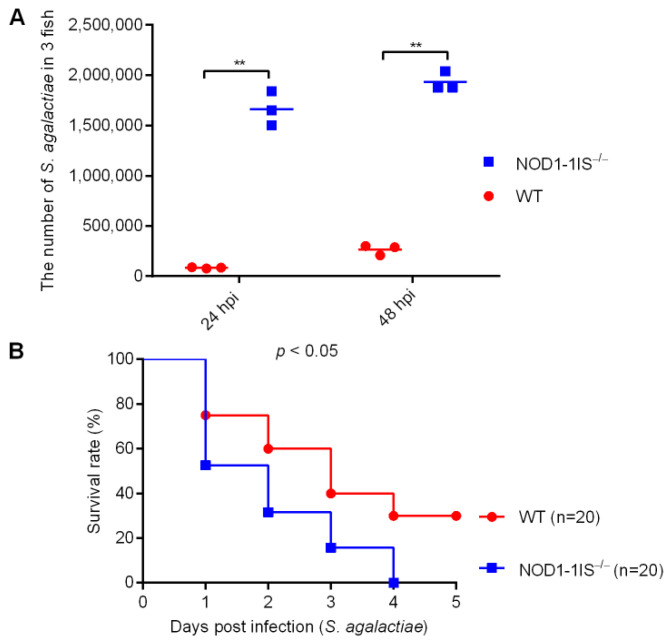
The effect of zebrafish NOD1 in *Streptococcus agalactiae* infection. (**A**) Effect of NOD1 knockdown on the proliferation of *S. agalactiae* in the mixture of liver and spleen from zebrafish at the age of 7 months. **, *p* < 0.01. (**B**) *NOD1^−/−^* zebrafish were more sensitive to *S. agalactiae* infection compared with the WT based on the survival rate.

Among NLRs, NLRP3 but not NOD2 plays a crucial role in the control of in vivo *S. agalactiae* growth and in host resistance against *S. agalactiae* infection. Zebrafish NOD1 is known to be involved in the host recognition of *Salmonella enterica* and spring viremia of carp virus [32,33], but its role in the host response to *S. agalactiae* infection has not been described. Previous studies have developed a zebrafish model of *S. agalactiae* infection by injection to study bacterial and host factors that contribute to disease progression [34,35]. Bath immersion can also be applied to adult zebrafish, but direct injection methods are more frequently used [35]. Therefore, to explore the effect of NOD1 in *S. agalactiae* infection, WT and *NOD1-1IS^−/−^* (NOD1*^−/−^* mutants with 1 bp insertion) adult zebrafish were infected intraperitoneally with *S. agalactiae* according to the method commonly used in streptococcal infections [35]. The bacterial burden in the mixed samples from the liver and spleen at different times post-infection was measured. Compared with WT adult zebrafish, significantly higher bacteria were detected in *NOD1-1IS^−/−^* adult zebrafish at 24 and 48 h post-infection (hpi) (Figure 1A). Infected zebrafish were monitored for mortality up to 5 days post-infection (dpi). Notably, *S. agalactiae*-infected *NOD1-1IS^−/−^* adult zebrafish exhibited reduced survival compared with the WT counterparts (Figure 1B). These data show that NOD1 protects against *S. agalactiae* infection.

### 2.2. NOD1 Deficiency Mainly Affects Metabolism and Immune System Processes in Adult Zebrafish in Response to S. agalactiae Infection

**Figure 2:**
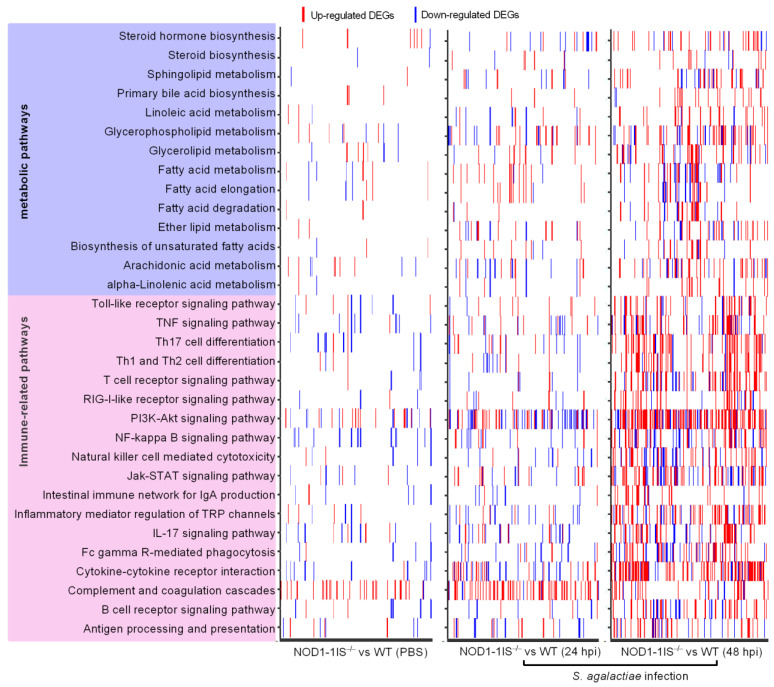
Heatmap presenting DEG profiles of each of the enriched immune-related and lipid metabolism pathways. The horizontal axis represents DEGs, and the vertical axis represents the enriched pathways. The existence of up-regulated DEG in a pathway is highlighted in red and down-regulated DEG is blue.

Our previous study revealed that the significantly enriched pathways were mainly involved in the metabolism and immune system for zebrafish larvae from WT and *NOD1-1IS^−/−^* collected at 10 dpf [29]. Signaling pathways regulated by NOD1 deficiency are still unclear in adult zebrafish in the case of bacterial infection. The systemic infection caused by the intraperitoneal injection was associated with the expansion of *S. agalactiae* in the blood, and *S. agalactiae* was found in the brain vasculature after 24 h post-injection [34]. As mutations or allele in mammalian NOD1 are known to be associated with susceptibility to inflammatory bowel disease (IBD), which is a chronic immune disorder of the intestine [36,37], the intestines from WT and *NOD1-1IS^−/−^* adult zebrafish were used for transcriptome sequencing to make clear the intestinal immune response regulated by NOD1 deficiency with or without *S. agalactiae* infection. The results showed that a total of 820 genes were considered to be differentially expressed genes (DEGs) for *NOD1-1IS^−/−^* vs. the WT group injected with PBS, 5407 DEGs at 24 hpi for *NOD1-1IS^−/−^* vs. the WT group infected with *S. agalactiae*, and 12,240 DEGs at 48 hpi for *NOD1-1IS^−/−^* vs. the WT group infected with *S. agalactiae*, respectively (Table 1). All of the DEGs for each group had an enrichment analysis of the KEGG pathways performed. The most DEGs were found at 48 hpi, and were mainly concentrated in the PI3K-Akt signaling pathway (154 DEGs), cytokine-cytokine receptor interaction (109 DEGs), Th17 cell differentiation (62 DEGs), and other immune-related signaling pathways. The second DEGs were found at 24 hpi, and were mainly concentrated in the complement and coagulation cascades (65 DEGs). The numbers of DEGs were the fewest for the *NOD1-1IS^−/−^* vs. WT group without bacterial infection, and were mainly concentrated in the complement and coagulation cascades (36 DEGs), NF-kappa B signaling pathway (16 DEGs), and other signaling pathways (Figure 2 and Supplementary File S1). Furthermore, many signaling pathways related to lipid metabolism, including Glycerophospholipid metabolism (41 DEGs), Glycerolipid metabolism (31 DEGs), Arachidonic acid metabolism (29 DEGs), Steroid hormone biosynthesis (27 DEGs), Sphingolipid metabolism (26 DEGs), Fatty acid degradation (22 DEGs), Ether lipid metabolism (21 DEGs), Primary bile acid biosynthesis (18 DEGs), Linoleic acid metabolism (18 DEGs), alpha-Linolenic acid metabolism (14 DEGs), Biosynthesis of unsaturated fatty acids (13 DEGs), and Steroid biosynthesis (11 DEGs), were significantly enriched at 48 hpi for the *NOD1-1IS^−/−^* vs. WT group infected with *S. agalactiae* (Figure 2 and Supplementary File S1).

**Figure 3:**
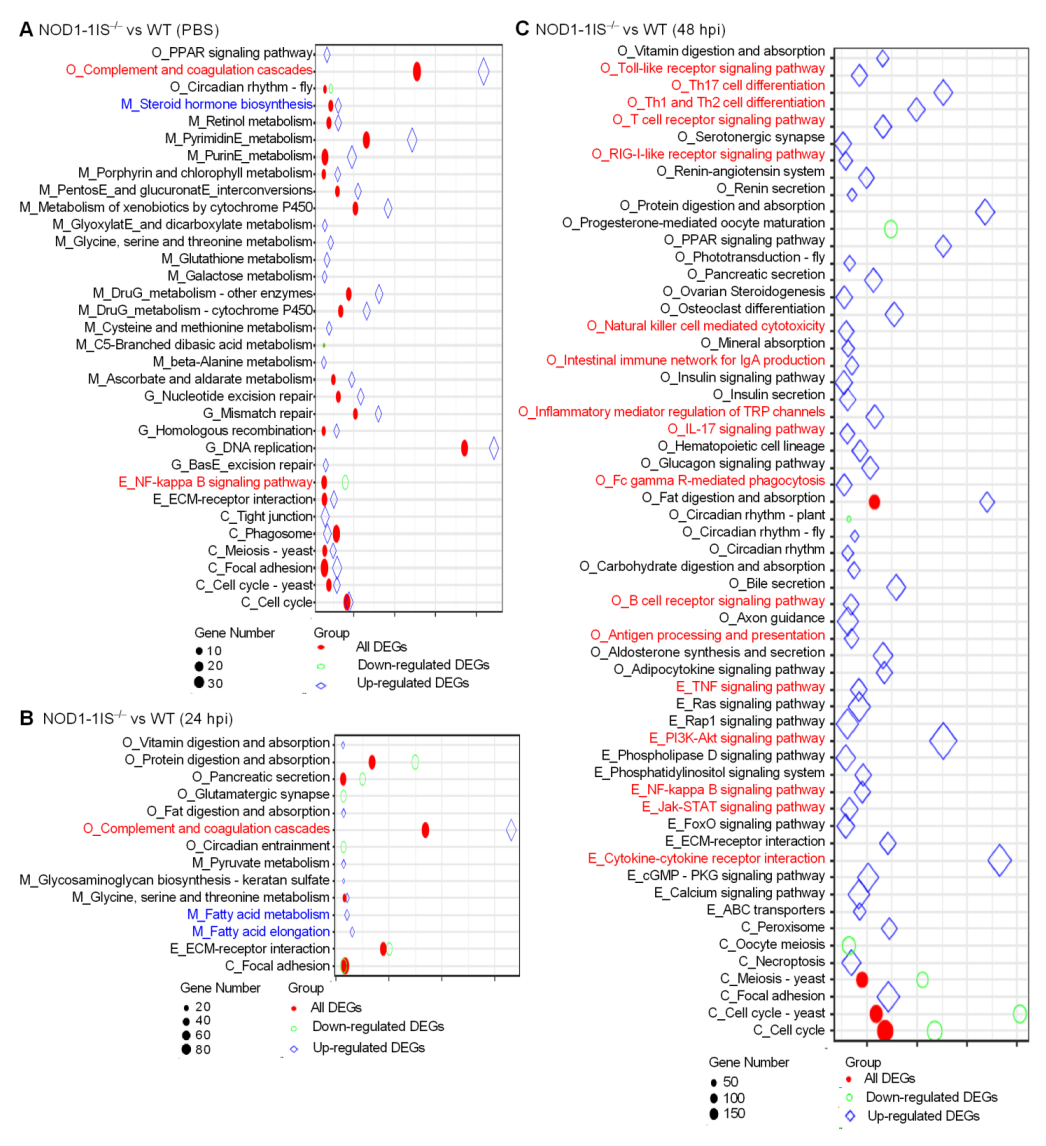
KEGG enrichment analysis. (**A**) The KEGG enrichment analysis for DEGs between WT and NOD1-deficiency zebrafish injected with the control PBS. (**B**) The KEGG enrichment analysis for DEGs between WT and NOD1-deficiency zebrafish infected with *S. agalactiae* for 24 h. (**C**) The KEGG enrichment analysis for DEGs between WT and NOD1-deficiency zebrafish infected with *S. agalactiae* for 48 h. All of the KEGG pathways shown in the figure are significantly enriched (Q-value < 0.05). The *x*-axis represents the rich factor, and the *y*-axis indicates the corresponding KEGG pathway. The immune-related pathways are red and the lipid-related pathways are blue. The color and shape of the point indicates the type of DEGs. The size of the point indicates the gene numbers.

A previous report characterized the transcription patterns of the intestines from WT adult zebrafish infected with *S. agalactiae.* A number of genes involved in the PI3K-Akt signaling pathway (125 DEGs), NOD-like receptor signaling pathway (65 DEGs), T cell receptor signaling pathway (50 DEGs), NF-kappa B signaling pathway (48 DEGs), Toll-like receptor signaling pathway (45 genes), B cell receptor signaling pathway (40 DEGs), and so on, were significantly downregulated at 48 hpi, which suggested the suppression of immune responses observed in the intestines and were consistent with the increasing mortality rate from 1 dpi to 4 dpi [38]. However, in the present study, NOD1 deficiency induced the expression of numerous DEGs involved in these immune signaling pathways in the case of *S. agalactiae* infection (Figure 3 and Supplementary File S1). Although many of the genes involved in the NOD-like receptor signaling pathway were regulated by NOD1 deficiency, the NOD-like receptor signaling pathway was not one of the significantly enriched pathways. The induced expressions of most immune-related DEGs in the intestines from *NOD1-1IS^−/−^* adult zebrafish seem very obscure for us at present, based on the fact that the reduced survival rate was observed in *NOD1-1IS^−/−^* adult zebrafish compared with WT counterparts with the *S. agalactiae* infection (Figure 1B). Different from the results in the intestines, many KEGG pathways, including Cytokine-cytokine receptor interaction (28 DEGs), Apoptosis (25 DEGs), IL-17 signaling pathway (19 DEGs), NF-kappa B signaling pathway (17 DEGs), and Hematopoietic cell lineage (17 DEGs), were significantly enriched for down-regulated DEGs in the skins from the same *NOD1-1IS^−/−^* adult zebrafish (Supplementary File S2). As the gut microbiota and its secreted metabolites contribute to regulating transcription, ROS modulation, and inflammation in the gut, they thereby play substantial roles in regulating the host metabolic and immune functions [39,40], which have been shown to act on both NOD1 and NOD2 receptors [41]. *S. agalactiae*-induced abnormal expression of most immune-related DEGs in the intestines from *NOD1-1IS^−/−^* adult zebrafish might be as a result of the outcome of immune imbalances regulated by NOD1 deficiency, the effects of intestinal microbiota and *S. agalactiae* infection in fatal conditions (100% mortality at 4 dpi). It is interesting to further investigate the effect of piscine NOD1 on the intestinal microbiota composition and activity with or without pathogen infection.

### 2.3. NOD1 Deficiency Regulated the Expression of Histone Variants and those Gene Variants Associated with Bile-Acid Signalling

**Figure 4:**
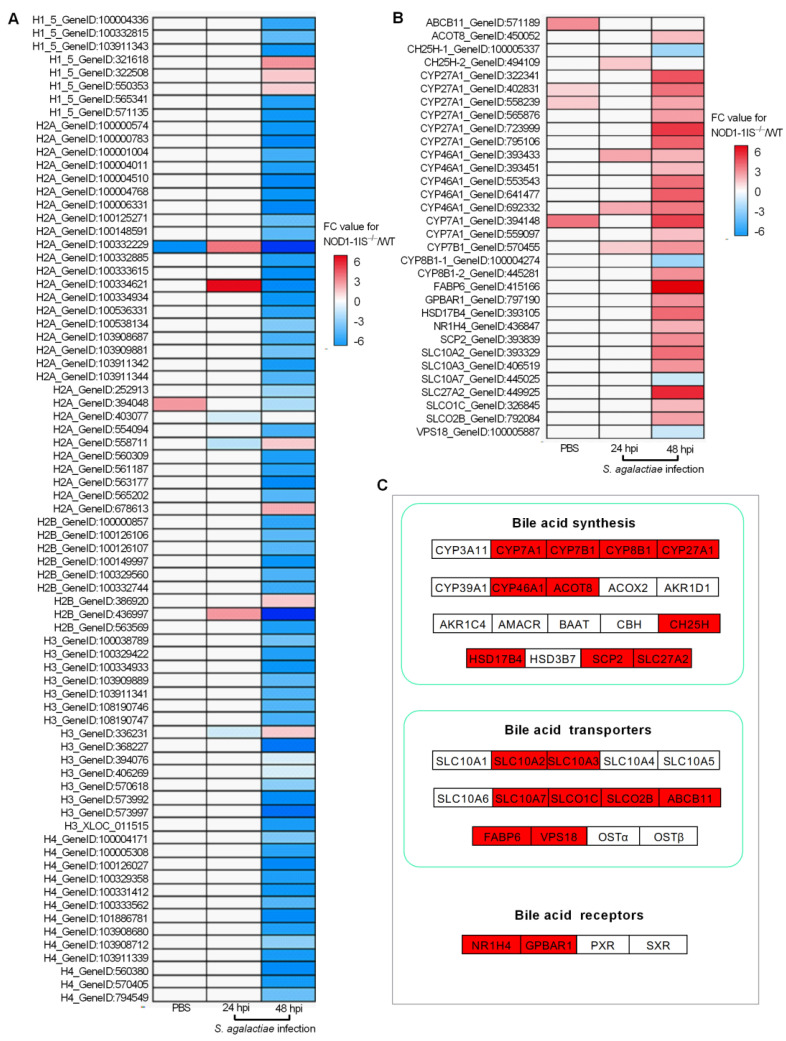
The effect of NOD1 deficiency on the transcriptional regulation of histones and many of the genes involved in bile acid metabolism. (**A**) Heatmap of differently expressed histones. (**B**) Heatmap of bile acid-related DEGs. (**C**) The expression trends of bile acid-related genes involved in bile acid synthesis, bile acid transporters, and bile acid receptors. The genes in red background were found to be differently expressed in the present study.

As RIP2 deficiency impairs the expression of histones [31], and NOD1 is also localized in the nucleus and is associated with chromatin [33], we are quite interested to know whether an interaction exists between NOD1 and histone. We firstly investigated the expression regulations of NOD1 deficiency on the histones through transcriptome analysis. Among the DEGs for *NOD1-1IS^−/−^* vs. WT group injected with PBS, two histone variants were identified. The numbers of histone variants were increased to 6 at 24 hpi for *NOD1-1IS^−/−^* vs. WT group infected with *S. agalactiae*, and 74 at 48 hpi for *NOD1-1IS^−/−^* vs. WT group infected with *S. agalactiae* (Table 1 and Figure 4A). Especially at 48 hpi for the *NOD1-1IS^−/−^* vs. WT group infected with *S. agalactiae*, 5 H1 variants, 27 H2A variants, 8 H2B variants, 14 H3 variants, and 13 H4 variants were significantly decreased by NOD1 deficiency (Figure 4A). All these data suggest that NOD1-RIP2 signaling regulated the transcription of histones.

Many studies have shown that bile acids and their receptors are involved in immune regulation of the host, in addition to playing a crucial role in glucose and lipid metabolism. Bile acids significantly regulate host immune function by activating G protein-coupled bile acid receptor 1 (TGR5, also known as *GPBAR1*) and FXR (namely *NR1H4*) receptors, causing changes in the host’s ability to resist bacteria, virus, and parasites [42,43,44]. Bile acids also activate the PKA-NLRP3 pathway through the TGR5 receptor, thereby improving the pathogenesis of inflammatory diseases including sepsis, celitis, and type 2 diabetes mellitus by inhibiting NLRP3 inflammasome [45]. Although the correlation between the NOD1 and bile acid metabolism has never been reported, research has shown that NOD2 deficiency resulted in an increased renal excretion of bile acids, which was mediated by an increased expression of the bile acid efflux transporters MRP2 and MRP4 in mice. This regulation of NOD2 deficiency on the hepatic bile acid concentration in turn significantly affected hepatocyte death and cholestatic liver disease [46]. Furthermore, it was suggested that bile acid receptors TGR5 and FXR might be therapeutic targets for digestive diseases and inflammatory diseases, including IBD [47,48,49,50,51]. Interestingly, in teleost, we demonstrated the conserved antiviral role of *GPBAR1* and its function in regulating glycerophospholipids metabolism [52]. In the present study, we found that 51, 117, and 282 lipid-related DEGs were regulated by NOD1 deficiency in the *NOD1-1IS^−/−^* vs. WT group injected with PBS, the *NOD1-1IS^−/−^* vs. WT group infected with *S. agalactiae* for 24 h, and the *NOD1-1IS^−/−^* vs. WT group infected with *S. agalactiae* for 48 h, respectively. Among them, 4, 4, and 30 DEGs involved in bile-acid signaling were regulated by NOD1 deficiency in *NOD1-1IS^−/−^* vs. WT group injected with PBS, *NOD1-1IS^−/−^* vs. WT group infected with *S. agalactiae* for 24 h, and *NOD1-1IS^−/−^* vs. WT group infected with *S. agalactiae* for 48 h, respectively (Table 1 and Figure 4B). Especially at 48 hpi for the *NOD1-1IS^−/−^* vs. WT group infected with *S. agalactiae*, 10 bile acid synthesis related genes, 8 bile acid transport related genes, and 2 bile acid receptor genes were found to be associated with NOD1 (Figure 4C). All of these results suggest that NOD1 may regulate bile acid metabolism and influence metabolic diseases via crosstalk with bile acid receptors such as *NR1H4* and *GPBAR1*. In future work, we would like to further study how the mutual regulation and interaction between NOD1 and bile acid receptors affect the occurrence of infectious diseases or metabolic diseases.

**Figure 5:**
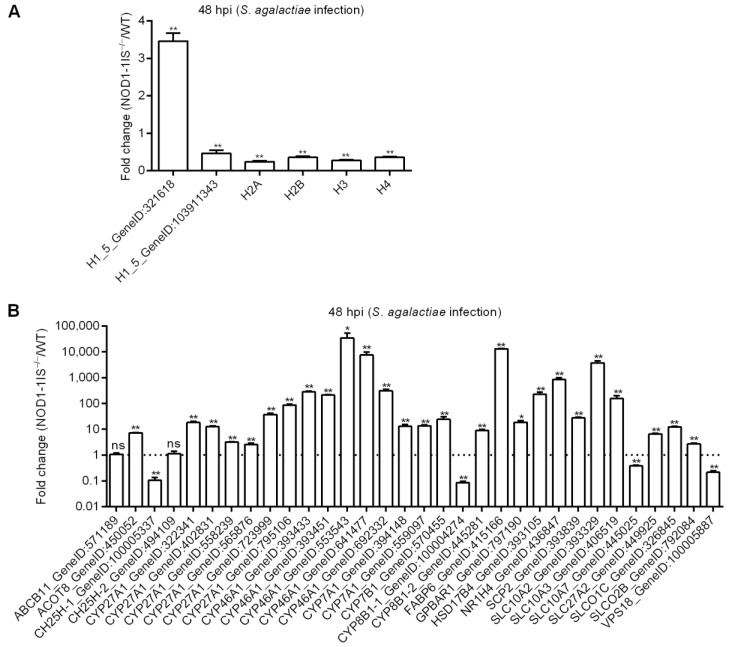
Validation of differential expression by qRT-PCR. (**A**) The expressions of candidate DEGs for histone variants confirmed by qRT-PCR. (**B**) The expressions of candidate DEGs for bile acid metabolism-related genes confirmed by qRT-PCR. Data represent the means ± the SEM and are tested for statistical significance using two-tailed student’s *t*-test. *, *p* < 0.05; **, *p* < 0.01; ns, not significant.

The expressions of 6 histones and 32 genes involved in bile acid metabolism were further confirmed by qRT-PCR in the *NOD1-1IS^−/−^* vs. WT groups infected with *S. agalactiae* for 48 h. Nucleotide polymorphism of H2A, H2B, H3, and H4 could not be distinguished by qRT-PCR, and the total expressions of H2A, H2B, H3, or H4 were detected by qRT-PCR (Figure 5A). At 48 hpi, the expression of all of the 38 genes tested was in agreement with the transcriptome data (Figure 5). Most histone genes were down-regulated by NOD1 deficiency (Figure 5A), however more genes involved in bile acid metabolism were up-regulated by NOD1 deficiency (Figure 5B). These results suggest that NOD1 deficiency does regulate the expressions of histones and bile acid metabolism-related genes.

### 2.4. Interaction Network Analysis of DEGs Related to Immunity and Metabolism Regulated by NOD1 Deficiency

WGCNA correlation analysis was performed on those DEGs related to immunity and metabolism. After ranking the edge weight of each connection, the first 500 edges were selected for visualization (all edges were selected for groups less than 500). The results showed that after screening, the lowest edge weight of the *NOD1-1IS^−/−^* vs. WT group without infection was 0.8307. The lowest edge weight of the *NOD1-1IS^−/−^* vs. WT group infected with *S. agalactiae* for 24 h was 0.9224, and was 0.9449 for the *NOD1-1IS^−/−^* vs. WT group infected with *S. agalactiae* for 48 h. These three groups contain 107, 89, and 172 nodes, respectively, and all these nodes belong to the same module.

**Figure 6:**
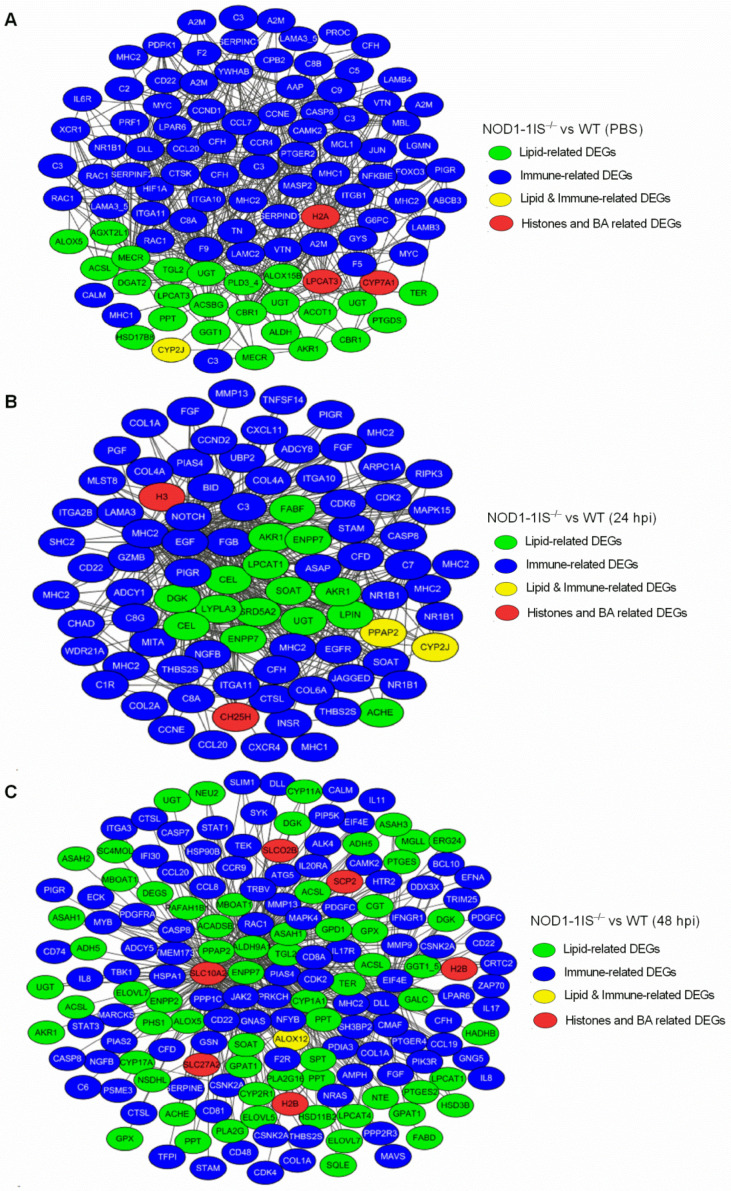
Co-occurrence network relationship among immune-related and metabolism-related DEGs via WGCNA analysis. (**A**) Co-occurrence network relationship among immune-related and metabolism-related DEGs in the *NOD1-1IS^−/−^* vs. WT groups injected with PBS. (**B**) Co-occurrence network relationship among immune-related and metabolism-related DEGs in *NOD1-1IS^−/−^* vs. WT group infected with *S. agalactiae* for 24 h. (**C**) Co-occurrence network relationship among immune-related and metabolism-related DEGs in the *NOD1-1IS^−/−^* vs. WT groups infected with *S. agalactiae* for 48 h. The node color indicates the biological function of each node.

The results of the WGCNA correlation analysis showed that some lipid metabolism genes are closely related to immune-related genes (Figure 6). More complement genes including C2, C3, C5, C8A, C8B, and C9 were found in the *NOD1-1IS^−/−^* vs. WT group without infection (Figure 6A). Relatively few complement genes, including C3, C7, C8A, and C8G, were found in the *NOD1-1IS^−/−^* vs. WT group infected with *S. agalactiae* for 24 h (Figure 6B). However, only C6 was found in the *NOD1-1IS^−/−^* vs. WT group infected with *S. agalactiae* for 48 h (Figure 6C). Histone H2A and bile acid synthesis gene CYP7A1 play important roles in the *NOD1-1IS^−/−^* vs. WT group without infection, but not critical in *NOD1-1IS^−/−^* vs. WT group infected with *S. agalactiae* for 24 h (Figure 6A,B). More DEGs related to lipid metabolism, including SLC10A2, SLC27A2, SLCO2B, SCP2, and so on, were found in the *NOD1-1IS^−/−^* vs. WT group infected with *S. agalactiae* for 48 h. Bile acid transport associated protein SLC10A2 and histone H2B were critical in the *NOD1-1IS^−/−^* vs. WT group infected with *S. agalactiae* for 48 h (Figure 6C). The WGCNA-derived “hub proteins” for H2A, H2B, H3, and DEGs related to lipid metabolism are beneficial for us to capture data for identifying novel histone binding complexes and reconstructing metabolic networks.

### 2.5. Histone H2A Is Translocated to the Cytoplasm and Colocalizes with NOD1 in the Case of S. agalactiae Infection

**Figure 7:**
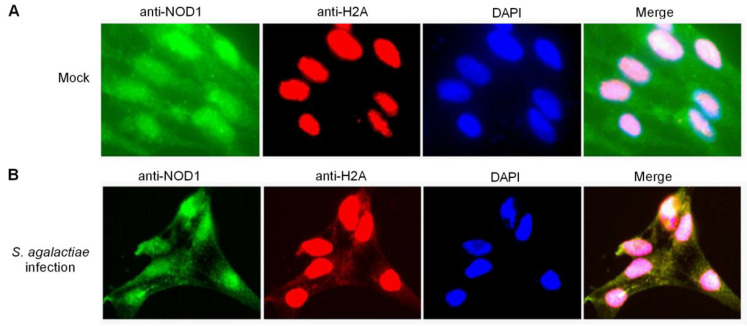
The colocalization between NOD1 and histone H2A in ZF4 cells. (**A**) The colocalization between NOD1 and histone H2A in mock-infected ZF4 cells. (**B**) The colocalization between NOD1 and histone H2A in the ZF4 cells infected with *S. agalactiae*.

Histones are synthesized in the cytoplasm, and rapidly imported into the nucleus by association with specific nuclear import receptors such as karyopherins (Kaps) or importins to promote their own nuclear localization [53,54,55]. However, after treatment with bleomycin, histone H1.2 was translocated from the nucleus to the mitochondria and co-localized with Bak in the mitochondria [56]. In apoptotic microglia, histone H3 did not stay concealed in the nucleus, and was found to leak from the nucleus into the cytoplasm [57]. In the present study, endogenous histone H2A was detected in the nucleus from the uninfected ZF4 cells, whereas no staining was observed in the cytoplasm using the anti-H2A antibody (Figure 7A). Endogenous histone H2A was distributed in the nucleus and cytoplasm in the ZF4 cells infected with *S. agalactiae* using anti-H2A antibody (Figure 7B). Furthermore, the merged images confirmed that NOD1 was colocalized with histone H2A both in the cytoplasm and cell nucleus in the case of *S. agalactiae* infection (Figure 7B). Although further studies are necessary to elucidate the regulatory mechanisms of histone H2A from the nucleus to the cytoplasm, our results suggest that NOD1 appears to be strongly linked with histone H2A.

### 2.6. NOD1 Interacts and Cooperates with Histone H2A Variant to Protect against S. agalactiae Infection

**Figure 8:**
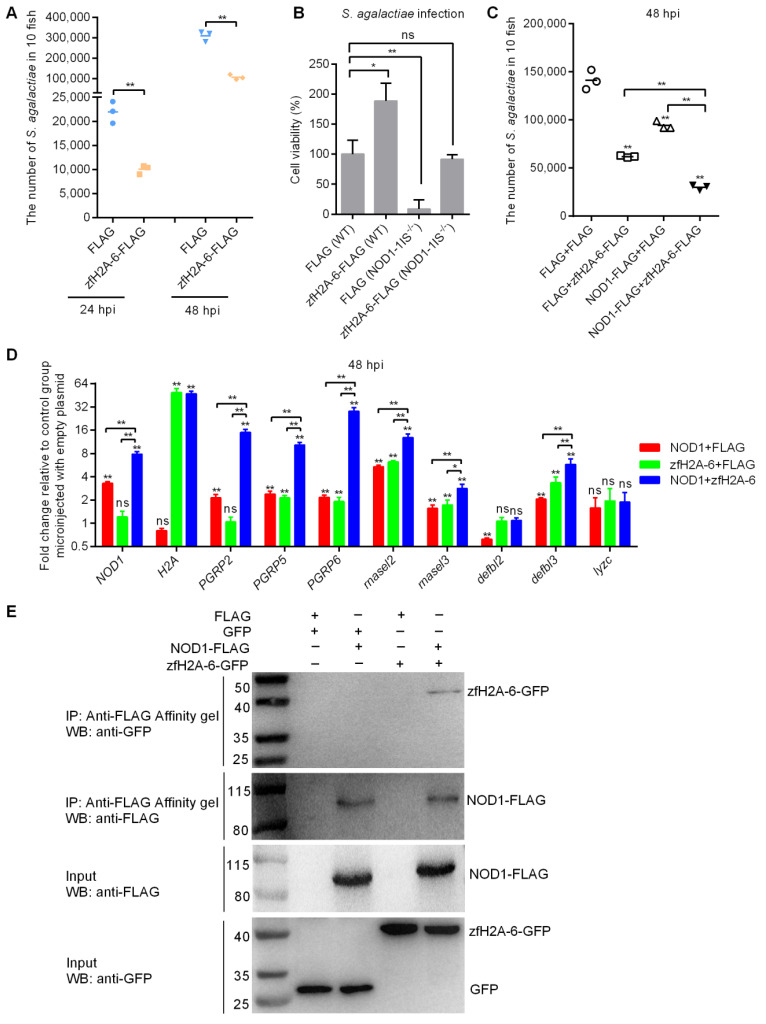
The effects of NOD1 and zfH2A-6 in the defense against *S. agalactiae* infection. (**A**) The effect of zfH2A-6 overexpression in the zebrafish larvae on the proliferation of *S. agalactiae.* (**B**) The effect of zfH2A-6 on the cell survival in WT and NOD1-deficient cells with *S. agalactiae* infection. (**C**) The effect of NOD1 on the antibacterial effect against *S. agalactiae* infection with or without the existence of zfH2A-6. (**D**) The transcription regulation of NOD1 on the antibacterial genes in the case of *S. agalactiae* infection with or without the existence of zfH2A-6. Data are expressed as mean ± SEM of three independent experiments. * *p* < 0.05; ** *p* < 0.01; ns—not significant. The asterisk above the error bars indicate statistical significance using the group transfected with empty plasmid as the control group. The asterisk above the bracket indicate statistical significance between the two groups connected by the bracket. (**E**) NOD1 interacts with zfH2A-6. The interaction between FLAG and GFP or zfH2A-6 is used as the negative controls.

Our previous study showed the antibacterial property of the complete histone H2A against Gram-negative bacteria *E. piscicida* [31]. We also found that nucleotide polymorphisms existed for zebrafish and grass carp H2A, and that nucleotide polymorphisms of piscine H2A significantly affected the antibacterial activities of piscine H2A [58]. In the present study, we selected a zebrafish histone H2A variant (zfH2A-6, GenBank accession number MT726195) with an antibacterial effect against *E. piscicida* infection for further study. Similar to the effect of zfH2A-6 in *E. piscicida* infection, the overexpression of zfH2A-6 significantly inhibited the bacteria proliferation of *S. agalactiae* in zebrafish larvae (Figure 8A). In the case of *S. agalactiae* infection, NOD1 deficiency enormously impaired cell survival, whereas the overexpression of zfH2A-6 in WT and NOD1-deficient cells promoted cell survival, which suggest that the effect of zfH2A-6 in promoting cell survival is independent of NOD1. Remarkably, the overexpression of zfH2A-6 in NOD1-deficient cells completely rescued the impaired cell survival caused by NOD1 deficiency (Figure 8B). As both NOD1 and zfH2A-6 significantly inhibited the bacteria proliferation of *S. agalactiae* and promoted the cell survival of infected cells, and that NOD1 deficiency decreased the expression of histone H2A, we concluded that the effect of NOD1 in the defense against *S. agalactiae* infection was associated with H2A. Furthermore, compared with the group alone transfected with NOD1 or zfH2A-6, the transient cotransfection of NOD1 and zfH2A-6 showed a cumulative antibacterial effect against *S. agalactiae* infection (Figure 8C), which indicated that the antibacterial activities of NOD1 and zfH2A-6 against *S. agalactiae* infection were not functionally redundant and antagonistic.

To further define the possible mechanisms in which zebrafish NOD1 and zfH2A-6 protect against *S. agalactiae* infection, the expressions of the antibacterial genes, including *PGRP2*, *PGRP5*, *PGRP6*, *defbl2*, *defbl3*, *rnasel2*, *rnasel3,* and *lyzc,* were examined by qRT-PCR. In the WT zebrafish with *S. agalactiae* infection, NOD1 overexpression induced the transcription of many antibacterial genes, including *NOD1* (3.2-fold), *PGRP2* (2.2-fold), *PGRP5* (2.4-fold), *PGRP6* (2.0-fold), *rnasel2* (5.1-fold), *rnasel 3* (1.4-fold), and *defbl3* (2.0-fold), and showed a decreased expression for *defbl2* and no significant change for *lyzc*, whereas zfH2A-6 overexpression significantly increased the transcription of *H2A* (46.2-fold), *PGRP5* (2.1-fold), *PGRP6* (1.8-fold), *rnasel2* (6.2-fold), *rnasel 3* (1.6-fold), and *defbl3* (3.6-fold). NOD1 together with zfH2A-6 synergistically increased the transcription of *NOD1* (7.5-fold), *PGRP2* (15.1-fold), *PGRP5* (10.1-fold), and *PGRP6* (25.1-fold), and cumulatively increased the transcription of *rnasel2* (11.2-fold), *rnasel 3* (2.7-fold), and *defbl3* (6.9-fold) (Figure 8D).

To check whether NOD1 physically interacts with the histone H2A variant, we performed co-immunoprecipitation in EPC cells. As shown in Figure 8E, no GFP (lane 1) and zfH2A-6 (lane 3) bands were observed, which confirmed that GFP and zfH2A-6 proteins were not pull-downed by FLAG. However, the pull-downed zfH2A-6 proteins by NOD1-FLAG (lane 4) were readily detected by immunoprecipitation analysis, which proved the interaction between NOD1 and zfH2A-6. Furthermore, previous studies have suggested that histones function as an important nuclear DAMP, and some TLRs contributed to histone recognition [59,60]. The interaction between NOD1 and zfH2A-6 in the present study may suggest that NOD1 is another receptor for histone recognition.

In mammals, CIITA and NLRC5 are well-known NLRs with transcriptional functions for inflammasome or MHC genes [61,62]. Our previous study showed that NOD1 was detected in the nucleus and chromatin of piscine and mammalian cells, and may have a regulatory function for gene transcription involved in the immune system [33]. The obvious co-localization and interaction of NOD1 and histone H2A, and the significant synergetic effect of NOD1 and the histone H2A variant in inducing the transcriptions of the upstream antibacterial PRRs may suggest that NOD1 and histone H2A have a strong positive feedback for regulating antibacterial signaling pathways in response to *S. agalactiae* infection.

## 3. Materials and Methods

### 3.1. Bacterial Infection for Adult Zebrafish and Sample Collection

Zebrafish wild-type AB/TU and *NOD-1IS^−/−^* mutants were obtained from the China Zebrafish Resource Center (CZRC), and were maintained in an aerated recirculation system at 28 °C with a photoperiod of light/dark (12 h/12 h). For bacterial infection of adult zebrafish, the healthy WT and *NOD1-1IS^−/−^* zebrafish at the age of 7 months were divided into two groups. Thirty WT or *NOD1-1IS^−/−^* zebrafish were intraperitoneally injected with 10 μL *S. agalactiae* (6.25 × 10^5^ cfu/μL), and another 30 WT or *NOD1-1IS^−/−^* zebrafish for 10 μL of PBS.

Nine fish from WT or *NOD1-1IS^−/−^* zebrafish injected with *S. agalactiae* or PBS (three fish for each sample and three samples for each group) were sacrificed at 24 and 48 hpi. Samples from the intestines and skins were flash-frozen in liquid nitrogen, and used for transcriptome sequencing. Samples from the intestines were also used for qRT-PCR verification. For the plate count or colony count, the mixtures from the liver and spleen were taken and lysed in 500 μL of PBS. The diluted bacterial suspension was plated onto BHI agar, then the CFUs were counted after 16 h of incubation at 37 °C.

Twenty fish for each group were used for the survival assay. The numbers of surviving zebrafish were counted daily for 5 days. GraphPad Prism 6 was used to generate survival curves, and the log-rank test was used to test differences in survival between the WT and **NOD1-1IS*^-/-^* zebrafish injected with *S. agalactiae*.

### 3.2. Bacterial Infection for Zebrafish Larvae and In Vivo Antibacterial Analysis of zfH2A-6 and/or NOD1

The p3 × FLAG-CMV-14 empty plasmid, NOD1-FLAG, or zfH2A-6-FLAG, were diluted to the desired concentration of 100 ng/μL. For the in vivo antibacterial assay of histone zfH2A-6, the p3 × FLAG-CMV-14 empty plasmid or zfH2A-6-FLAG were microinjected into fertilized eggs at the one-cell stage. The typical injected volume was 2 nl. At 4 days after fertilization, the hatched larvae microinjected with p3×FLAG-CMV-14 or zfH2A-6-FLAG were infected with 2 × 10^8^ CFU/mL *S. agalactiae.* Ten larvae per group were collected at 24 and 48 hpi, and were rinsed and lysed in 1 mL of PBS. The diluted homogenates were plated onto BHI agar, and the CFUs were counted after 12 h of incubation at 37 °C.

For the cumulative antibacterial effect of NOD1 and histone zfH2A-6, the plasmids of p3 × FLAG-CMV-14, zfH2A-6-FLAG, and NOD1-FLAG were microinjected with combination constructs of two plasmids (1:1) into one-stage embryos. At 4 days after fertilization, the hatched larvae microinjected with indicated plasmids were infected with 2 × 10^8^ CFU/mL *S. agalactiae.* Ten larvae per group were collected at 48 hpi, and were rinsed and lysed in 1 mL of PBS. The diluted homogenates were plated onto BHI agar, and the CFUs were counted after 12 h of incubation at 37 °C.

### 3.3. Bacterial Infection for Zebrafish WT and NOD1*^−/−^* Cell Lines and Cell Counting kit-8 (CCK-8) Assay

Primary cell cultures were developed from caudal fins of WT and *NOD1-1IS^−/−^* mutants at the age of 1 month, and were successfully subcultured to the stable cell lines designated as WT or NOD1*^−/−^* caudal fin-derived cell lines by the tissue block adherent method. The cells beyond 50 passages were used for the CCK-8 assay (Beyotime, Wuhan, China). Approximately 10^4^ WT or NOD1*^−/−^* caudal fin-derived cells were seeded overnight in 96-well plates, and then infected with *S. agalactiae* with the MOI of 1 after 24 h. At 6 hpi, 10 μL CCK-8 reagent was added to a 90 μL DMEM/F12 culture medium to generate a working solution, of which 100 μL was added per well and was incubated for 2 h. The absorbance at 450 nm was measured by a PerkinElmer’s EnSpire Multilabel Plate Reader.

### 3.4. cDNA Library Construction and Illumina Deep Sequencing

The total RNA was isolated from the intestines or skins of the WT and *NOD1-1IS^−/−^* zebrafish injected with PBS or *S. agalactiae* at 24 hpi and 48 hpi using TRIzol^®^ Reagent (Invitrogen) according to the manufacturer’s introduction. RNA integrity was assessed using an Agilent 2100 bioanalyzer (Agilent, Santa Clara, CA, USA). Samples with RNA integrity numbers (RINs) ≥7.5 were subjected to cDNA library construction using the TruseqTM RNA sample prep Kit (Illumina). To identify the DEGs between the WT and *NOD1-1IS^−/−^* adult zebrafish, the expression levels were measured using numbers of fragments per kilobase of transcript per million fragments sequenced (FPKM), and were identified based on FDR (false discovery rate) <0.05, log_2_FC (fold change (condition 2/condition 1) for a gene) >1 or log_2_FC < −1. The significantly enriched pathways of these DEGs were determined using the KEGG (Kyoto Encyclopedia of Genes and Genomes) database. Bonferroni correction was used to adjust *p*-values, and KEGG pathways with adjusted *p* value (Q-Value) < 0.05 were considered significantly enriched. The FPKMs of DEGs were used for the co-expression network analysis using the WGCNA packages in R [63]. The edge weights were sorted from the highest to the lowest, and the network with the top 500 edges was visualized using Cytoscape_v.3.6.1 [64].

### 3.5. qRT-PCR Validation of DEGs

qRT-PCR analysis was performed to validate the candidate DEGs for transcriptome samples at 48 hpi under the following conditions: 3 min at 95 °C, followed by 45 cycles of 15 s at 94 °C, 15 s at 54~58 °C, and 30 s at 72 °C. All of the reactions were performed in triplicate in a 96 well plate and the mean value was recorded. The DEGs for validation included H1.1 (NM_199552.1, GeneID:321618), H1.2 (XM_009302911.3, GeneID:103911343), ABCB11 (XM_003199465.5, GeneID:571189), ACOT8 (NM_001006072.1, GeneID:450052), CH25H(XM_021476542.1, GeneID:100005337), CH25H(NM_001008652.1, GeneID:494109), CYP27A1 (NM_001328513.1, GeneID:322341), CYP27A1 (XM_002663399.5, GeneID:402831), CYP27A1 (XM_681430.7, GeneID:558239), CYP27A1 (NM_001123277.1, GeneID:565876), CYP27A1 (XM_009304846.3, GeneID:723999), CYP27A1 (XM_001333968.7, GeneID:795106), CYP46A1 (NM_200461.1, GeneID:393433), CYP46A1 (NM_200479.2, GeneID:393451), CYP46A1 (NM_001020522.1, GeneID:553543), CYP46A1 (NM_001037418.1, GeneID:641477), CYP46A1 (NM_001045298.1, GeneID:692332), CYP7A1 (NM_201173.2, GeneID:394148), CYP7A1 (XM_682404.6, GeneID:559097), CYP7B1 (XM_693936.8, GeneID:570455), CYP8B1 (NM_001110288.1, GeneID:100004274), CYP8B1 (NM_001003736.1, GeneID:445281), FABP6 (NM_001002076.2, GeneID:415166), GPBAR1 (XM_017357898.2, GeneID:797190), HSD17B4 (NM_200136.1, GeneID:393105), NR1H4 (NM_001002574.1, GeneID:436847), SCP2 (NM_200865.2, GeneID:393839), SLC10A2 (NM_200358.1, GeneID:393329), SLC10A3 (NM_214740.1, GeneID:406519), SLC10A7 (NM_001003420.1, GeneID:445025), SLC27A2 (NM_001025299.1, GeneID:449925), SLCO1C (NM_001348086.1, GeneID:326845), SLCO2B (NM_001037678.2, GeneID:792084), and VSP18 (NM_173245.2, GeneID:100005887). The housekeeping gene GAPDH was used for normalizing the cDNA amounts. The primers specific for the interested DEGs are listed in Supplementary Table S1.

### 3.6. qRT-PCR Analysis of Antibacterial Genes Regulated by NOD1 and/or zfH2A-6

The plasmids of p3×FLAG-CMV-14, zfH2A-6-FLAG, and NOD1-FLAG at a concentration of 100 ng/μL were microinjected with the indicated combination constructs of two plasmids (1:1) into one-stage embryos. At 4 days after fertilization, the hatched larvae were infected with 2 × 10^8^ CFU/mL *S. agalactiae.* Fifty larvae per group were collected at 48 hpi, and were used for RNA extraction and qRT-PCR using primers specific to the antibacterial genes, including *PGRP2*, *PGRP5*, *PGRP6*, *defbl2*, *defbl3*, *rnasel2*, *rnasel3,* and *lyzc*. The primer sequences used for qRT-PCR are described by our previous studies [31,65]. The housekeeping gene GAPDH was used for normalizing the cDNA amounts.

### 3.7. Fluorescence Microscopy

ZF4 cells plated overnight onto coverslips in 12-well plates were infected with *S. agalactiae* with a MOI of 1. At 24 hpi, the cells were washed three times with PBS, fixed for 1h at room temperature by 4% PFA, incubated overnight with the primary antibodies including anti-NOD1 [24] and anti-H2A (catalog no. 12349S, CST), incubated for 1 h with fluorescent labeled secondary antibodies (catalog no. R37121 and A11029; Thermo, Waltham, MA, USA), and finally stained with DAPI (1 μg/mL) for 15 min. The coverslips were observed with a confocal microscope (SP8; Lecia, Wetzlar, Germany).

### 3.8. Co-Immunoprecipitation (Co-IP) and Western Blotting

To test the interaction between NOD1 and zfH2A-6, the EPC cells were co-transfected with the FLAG empty plasmid, GFP empty plasmid, NOD1-FLAG, or zfH2A-6-GFP with the indicated combinations of two plasmids (1:1). At 48 h post-transfection, the cells were washed with ice-cold PBS three times and then lysed in a Pierce™ IP lysis buffer (Thermo Scientific™, Waltham, MA, USA #87787) containing Protease Inhibitor Cocktail (Thermo Scientific™, #78430). Co-IP was performed using a FLAG Tagged Protein Immunoprecipitation Kit (Sigma, St. Louis, MO, USA) according to the manufacturer’s manual. The total lysate and eluted proteins were analyzed by Western blotting using monoclonal mouse anti-FLAG antibody (Sigma, St. Louis, MO, USA, F3165) and anti-TurboGFP polyclonal antibody (Evrogen, Moscow, Russia, CAT. # AB513).

### 3.9. Statistical Analysis

Significance testing in the cumulative survival analysis used log-rank test in GraphPad Prism 6. Expression data by qRT-PCR are presented as means and standard error of mean (SEM). Two-tailed Student’s t-test or ANOVA were used to compare the means and SEM between groups. All of the data are representative of three biologic replications. The level of significance is shown as follows: * *p* < 0.05; ** *p* < 0.01.

## 4. Conclusions

*S. agalactiae* is a kind of Gram-positive bacteria that exists widely in the natural world, and can cause enormous economic losses both for saltwater and farmed freshwater fish. In the present study, we firstly reported that NOD1 and histone H2A variant contribute to host defense against *S. agalactiae* infection in teleost. The transcriptional regulations of NOD1 deficiency on the histone variants and those gene variants associated with bile-acid signaling were characterized, along with immune-related and metabolic pathways regulated by NOD1 in response to *S. agalactiae* infection. Furthermore, the colocalization, interaction, and functional correlation between NOD1 and the histone H2A variant in the defense against *S. agalactiae* infection were confirmed. Further studies are required to address how the crosstalks between NOD1 and other histone variants or bile acid receptors affect the occurrence of infectious diseases and/or metabolic diseases.

## Figures and Tables

**Table 1:** Summary of RNAseq profiling in *NOD1-1IS^−/−^* vs. WT adult zebrafish without or with *S. agalactiae* infection.

Group	Total DEGs	Immune-Related DEGs	Lipid-Related DEGs	Histone Variants	Bile Acid-Related DEGs
*NOD1-1IS^−/−^* vs. WT (PBS)	820	135	51	2	4
*NOD1-1IS^−/−^* vs. WT (24 hpi)	5407	340	117	6	4
*NOD1-1IS^−/−^* vs. WT (48 hpi)	12,240	719	282	74	30

## Data Availability

The data is available in this manuscript.

## Supplementary Materials

The following are available online at https://www.mdpi.com/article/10.3390/antibiotics10070861/s1, File S1: The significantly enriched pathways in intestines, File S2: The significantly enriched pathways in skins, Table S1: Primer information.
