# Supplementary material for: Crosstalks between NOD1 and Histone H2A Contribute to Host Defense against Streptococcus agalactiae Infection in Zebrafish
Source: Antibiotics (Basel). 2021 Jul 15;10(7):861. doi: 10.3390/antibiotics10070861 (PMC8300774; doi:10.3390/antibiotics10070861)
Supplement: Supplementary file 1 [file antibiotics-10-00861-s001.zip › Supplementary Table S1.pdf]

Table S1 Primer information

Primer Name	Primer Sequences (5' to 3')	Application
zfH2A-6F	GTCAAGCTTAAGACCAAGATGAGCGGAA	Ligated to p3xFLAG-CMV™-14 vector
zfH2A-6R	GATGGTACCTTGCCTTTGGCAGCCTTCTC	
NOD1F	GTGGAATTCTGAAATGAAATTAAATATGG	
NOD1R	GAAGGATCCGCTGACTCCCTCTCGTTG	
H1_5_GeneID:321618F	AGTCGGAGCTGTCCAAGAAA	Quantitative real-time PCR
H1_5_GeneID:321618R	GTTGTCGCCCACCTTGTAGT	
H1_5_GeneID:103911343F	CATCTGTGAAGAAGGCGACA	
H1_5_GeneID:103911343R	AAAAGAGCCGTTTGGGTTTT	
H2AF	ACGCCGCTCGGGACAACAAGAAG	
H2AR	GCGATGGTCACTCCACCCAGAAG	
H2BF	CGGTAAGGGAGGAAAGAAGCG	
H2BR	TGTAGTGAGCGAGACAAGACG	
H3F	GTGAGATCCGCCGCTACCAGAAG	
H3R	GTGTCCTCGAACAGACCAACCAG	
H4F	CAAAGGCGGTAAAGGTCTTGGAAA	
H4R	TCTTGGCGTGCTCGGTGTATGTAA	
ABCB11_GeneID:571189F	CGGCTCCAGTAGCTCGTATC	
ABCB11_GeneID:571189R	AGGATCTGGCATCGAAAATG	
ACOT8_GeneID:450052F	CAGCGCTTCCTCAGTAAACC	
ACOT8_GeneID:450052F	GCAGTTTCATATCGCCAGGT	
CH25H_GeneID:494109F	TCCATAAGGTGCACCACAAA	
CH25H_GeneID:494109R	GTTCAGCATGTGGAAGAGCA	
CH25H_GeneID:100005337F	GGATGTGTTCAGTCCACACG	
CH25H_GeneID:100005337R	GCAAACGTTTCCCTGTTCAT	
CYP27A1_GeneID:322341F	GGCCACAGAAAGCTGAAGAC	
CYP27A1_GeneID:322341R	ACTGAAGCGAGTCCTTTGGA	
CYP27A1_GeneID:402831F	GGCCACAGAAAGCTGAAGAC	
CYP27A1_GeneID:402831R	TCTCCCCTGGAAGGAAACTT	
CYP27A1_GeneID:558239F	CTACCGGGGCCTCTTAAAAC	
CYP27A1_GeneID:558239R	AGACTTCAGGGTTCGAGCAA	
CYP27A1_GeneID:565876F	CTGGATCCGAGAGCAGAAAC	
CYP27A1_GeneID:565876R	GACGCATTTGCTTTGACAGA	
CYP27A1_GeneID:723999F	CAACTGGACCCGCAATTACT	
CYP27A1_GeneID:723999R	GCACCACCGGATACAGTCTT	
CYP27A1_GeneID:795106F	GGCCACAGAAAGCTGAAGAC	
CYP27A1_GeneID:795106R	GAACCGAGTCCTTTGGATGA	
CYP46A1_GeneID:393433F	GATGAAGTCCTCGGAACCAA	
CYP46A1_GeneID:393433R	GACAGGTTCGTGGACCAAGT	
CYP46A1_GeneID:393451F	GATGAAGTCCTCGGAACCAA	
CYP46A1_GeneID:393451R	GACAGGTTCGTGGACCAAGT	
CYP46A1_GeneID:553543F	ACTTGGTCCACGAACCTGTC	

CYP46A1_GeneID:553543R	GCGCCGTTACAACCTACATT	
CYP46A1_GeneID:641477F	ATCATGGATCCAGCCTTCAG	
CYP46A1_GeneID:641477R	ACGCAGGAGTTCAGTTGCTT	
CYP46A1_GeneID:692332F	AACAGCCAATCAGCTCTCGT	
CYP46A1_GeneID:692332R	GGCGCATTCACATTAAACCT	
CYP7A1_GeneID:394148F	TTCACAAGCCAACACTCTGC	
CYP7A1_GeneID:394148R	TCATCGGTGGGTACATAGCA	
CYP7A1_GeneID:559097F	CTTCATCGGCGGAAATGTAT	
CYP7A1_GeneID:559097R	AGCAGCTGTTTGATCGGACT	
CYP7B1_GeneID:570455F	CCGAACAGCTCTTTTTCAGG	
CYP7B1_GeneID:570455R	TTTTTCTCTCCGAGCACGTT	
CYP8B1_GeneID:100004274F	CAGCGAATGAAAGCAAAACA	
CYP8B1_GeneID:100004274R	ACACTTTGCCATTCCCAGAC	
CYP8B1_GeneID:445281F	GTGGTCAAAGAGGCAAGAGC	
CYP8B1_GeneID:445281R	ACACTTTGCCATTCCCAGAC	
FABP6_GeneID:415166F	CGTCCAGAACGGAGATGACT	
FABP6_GeneID:415166R	TTGGGGAAGCTTATGGTCAG	
GPBAR1_GeneID:797190F	CGACAGCTGGATTTCAGACA	
GPBAR1_GeneID:797190R	AGGCTTTGGGAAAAACCTGT	
HSD17B4_GeneID:393105F	GAACCACATGAAGCAGCAGA	
HSD17B4_GeneID:393105R	CGTACTCCGCCTTCAGAGAC	
NR1H4_GeneID:436847F	CACAACAAACATCGCATTCC	
NR1H4_GeneID:436847R	GCTGAAGACTTGGGCTGAAC	
SCP2_GeneID:393839F	AGGCGGTAGAGATCGTAGCA	
SCP2_GeneID:393839R	ATTGGCAGAGAAGCAGTCGT	
SLC10A2_GeneID:393329F	TGAGTTTGCTTGTGCCTGTC	
SLC10A2_GeneID:393329R	GCCACAACCAGTTGGAAGAT	
SLC10A3_GeneID:406519F	TGATGAGTGCAGGAGTGGAG	
SLC10A3_GeneID:406519R	AGGATGGAGATGACGTCTGG	
SLC10A7_GeneID:445025F	AACCAACGAGACCAAACCAG	
SLC10A7_GeneID:445025R	CAATACTCGGGTCGTCAGGT	
SLC27A2_GeneID:449925F	ACACTTACCGGGATGCAGAC	
SLC27A2_GeneID:449925R	GTCTTGTGTCTCCGCCTCTC	
SLCO1C_GeneID:326845F	GTAACGGGTGGAGTGATGCT	
SLCO1C_GeneID:326845R	GGCAGGAGGATTGTGTTTGT	
SLCO2B_GeneID:792084F	GACTCAACTCCAGCCTTTGC	
SLCO2B_GeneID:792084R	TCGACATACAAGCGAAGCAC	
VPS18_GeneID:100005887F	CTGGAGGTTGAACGTGGTTT	
VPS18_GeneID:100005887R	GCAGGAGCAAGAAGTGGAAC	
